# Dysphagia in patients with severe stroke: influencing factors and coping strategies

**DOI:** 10.3389/fneur.2026.1767339

**Published:** 2026-06-17

**Authors:** Sulian Gu, Yu Chen, Yue Cui, Ling Yu

**Affiliations:** Department of Neurology, The First Affiliated Hospital with Nanjing Medical University, Nanjing, Jiangsu Province, China

**Keywords:** care, clinical, dysphagia, neurology, nursing, stroke

## Abstract

**Background:**

Dysphagia is a common and serious complication in patients with severe stroke. This study aimed to identify independent risk factors for dysphagia in this population, construct a predictive model, and provide evidence-based support for risk stratification.

**Methods:**

A retrospective cohort study was conducted on patients with severe stroke admitted to the Neurointensive Care Unit (NICU) of a Grade A tertiary hospital from January 2023 to August 2025. Patients were divided into a dysphagia group (*n* = 288) and a non-dysphagia group (*n* = 70) based on swallowing assessment. Clinical data were collected and compared.

**Results:**

Among 358 patients with severe stroke, the incidence of dysphagia was 80.45%. Logistic regression identified age ≥70 years (OR = 6.452, 95%CI: 3.825–10.936), brainstem/basal ganglia lesion location (OR = 4.608, 95%CI: 2.751–7.723), GCS score ≤8 points (OR = 8.654, 95%CI: 5.012–14.928), mechanical ventilation use (OR = 6.863, 95%CI: 4.015–11.729), and sedative use (OR = 5.382, 95%CI: 3.156–9.187) as independent risk factors. A predictive model based on these five factors yielded an area under the ROC curve of 0.864 (95%CI: 0.812–0.916, *p* < 0.001).

**Conclusion:**

Dysphagia occurs in over 80% of patients with severe stroke. The proposed model, using readily available clinical variables, shows potential for early risk identification. However, external validation in prospective multicenter cohorts is required before routine clinical application.

## Introduction

Severe stroke is one of the most common acute and critical illnesses in the Neurointensive Care Unit (NICU), characterized by abrupt onset, severe neurological impairment, significant disease fluctuation, multiple complications, and poor prognosis. In this study, severe stroke was defined as stroke requiring NICU admission, with a National Institutes of Health Stroke Scale (NIHSS) score >15 or a Glasgow Coma Scale (GCS) score ≤12, reflecting the high acuity and intensive care needs of this population. Its risks of disability and mortality are significantly higher than those of general stroke patients (1, 2). For example, general stroke patients have a 30-day mortality rate of approximately 5–10%, whereas among severe stroke patients requiring ICU admission, the 30-day mortality rate for severe ischemic stroke is approximately 27%, and that for intracerebral hemorrhage can reach 41% (3), highlighting a strong association between disease severity and early death. Based on large-scale critical care databases and cohort studies, the short-term mortality rate of stroke patients requiring ICU admission/receiving intensive care remains at a relatively high level: among ICU inpatients, the 30-day mortality rate of severe ischemic stroke is approximately 27%, and that of intracerebral hemorrhage patients can reach 41% (4), indicating a strong correlation between disease severity and early mortality risk. With the acceleration of population aging and advances in reperfusion therapy, life support, and neurocritical care management, the survival rate of severe stroke patients has improved (5). However, survivors often suffer from severe consciousness disorders and multidimensional functional deficits, and frequently require therapeutic support such as mechanical ventilation and sedation-analgesia, which further increase the risks of multisystem complications and nursing challenges (6). Therefore, early identification and precise intervention targeting key complications of severe stroke are crucial for reducing mortality, shortening hospital stays, and improving long-term functional outcomes (7, 8).

Dysphagia is one of the most common complications after stroke and has the most significant impact on clinical outcomes, with a higher incidence and longer duration in severe stroke patients (9). Systematic reviews (10, 11) have shown that the overall prevalence of post-stroke dysphagia in the acute phase is approximately 42%, and can be as high as 50–80% across different populations and assessment methods (12). Moreover, the severity of stroke and the degree of impaired consciousness are positively associated with the risk of dysphagia. Impaired swallowing function can lead to overt or occult aspiration, which in turn induces severe adverse events such as aspiration pneumonia, asphyxia, malnutrition, and dehydration (13). A meta-analysis indicated that stroke patients with dysphagia have a significantly increased risk of developing pneumonia compared to those without dysphagia (odds ratio [OR] ≈ 9.6) (14). Clinical practice further demonstrates that the development of dysphagia is multifactorial, involving interactions between age, airway management, sedative use, and other factors (9, 15). Delayed screening and nursing intervention can easily lead to a vicious cycle of “aspiration—infection—re-injury,” which significantly impairs rehabilitation outcomes and increases the burden of critical care (16, 17).

Based on the above background, this study aims to systematically explore the influencing factors of dysphagia in severe stroke patients, identify the characteristics of high-risk populations and modifiable intervention points, and propose operable risk assessment and nursing strategies. The goal is to achieve early prediction, precise prevention, and standardized management of dysphagia, thereby improving the overall prognosis and quality of life of patients with severe stroke.

## Methods

### Ethical approval

This study was reviewed and approved by the Medical Ethics Committee of our hospital (Ethics Approval No.: 2024-SR-100) and strictly adhered to the Declaration of Helsinki (18). As a retrospective analysis, all data were extracted from the hospital’s electronic medical record system. During data extraction, personally identifiable information such as patients’ names, hospital admission numbers, and ID card numbers was fully anonymized, with only objective clinical diagnosis and treatment-related data retained to maximize the protection of patients’ privacy rights and interests. Since the study did not involve direct intervention in patients’ diagnosis and treatment processes and all data were routinely collected clinical information, the informed consent procedure for patients was waived after review by the Ethics Committee.

### Study design

A retrospective cohort study design was adopted in this study. The study subjects were patients with severe stroke admitted to the NICU of a Grade A tertiary hospital in China. Clinical baseline data, disease diagnosis and treatment information, and swallowing function assessment results of patients were systematically extracted from the hospital’s electronic medical record system, nursing record system, and laboratory examination database. According to the presence or absence of dysphagia during hospitalization, the study subjects were divided into the dysphagia group and the non-dysphagia group. The distribution differences of candidate influencing factors between the two groups were compared and analyzed to identify the independent risk factors for dysphagia in patients with severe stroke, thereby providing evidence-based basis for the clinical development of targeted nursing intervention strategies. Strict quality control was implemented throughout the study: two uniformly trained researchers independently extracted data, which were cross-checked after extraction. In case of data discrepancies, a consensus was reached by jointly reviewing the original medical records or consulting senior neurologists to ensure the accuracy and completeness of the data.

### Sample size calculation

The sample size was estimated based on the principle of sample size determination for multivariate Logistic regression analysis (19), i.e., each independent variable included in the model should correspond to at least 10–15 outcome events (the outcome event in this study was the occurrence of dysphagia). A total of 11 candidate influencing factors were included in this study. Combined with preliminary clinical practice data and relevant literature reports (12, 20), the incidence of dysphagia in patients with severe stroke was estimated to be approximately 80%. PASS 15.0 statistical software was used for sample size calculation: the significance level *α* = 0.05 (two-tailed test), power 1 − *β* = 0.90, and allowable error *δ* = 0.15 were set. The calculated minimum required sample size was 320 cases. Considering the possible missing clinical data and exclusion of unqualified cases, and based on our institution’s historical missing data rate (approximately 10–15%), the sample size was further expanded by approximately 10–15% to ensure the study power. Finally, 358 patients were included, which met the sample size requirements for multivariate Logistic regression analysis and subsequent model validation, ensuring the reliability and statistical stability of the study results.

### Study population

From January 2023 to August 2025, a total of 452 consecutive patients with severe stroke admitted to the NICU were screened. After applying the inclusion and exclusion criteria, 94 patients were excluded for the following reasons: did not meet the requirement of NICU stay ≥72 h or onset-to-admission time ≤24 h (*n* = 20); age <18 years (*n* = 2); pre-existing dysphagia of any etiology (*n* = 8, as described below); severe comorbidities independently confounding swallowing assessment, such as advanced liver failure or active malignancy (*n* = 12); voluntary discharge, transfer, or death before swallowing assessment could be performed (*n* = 10); incomplete clinical data with missing key variables (e.g., GCS score, lesion location) (*n* = 24); and severe cognitive impairment or psychiatric illness precluding cooperation with required evaluations (*n* = 18). The remaining 358 patients constituted the final study cohort.

Inclusion criteria: (1) Met the diagnostic criteria for severe stroke as defined in the Chinese Guidelines for the Diagnosis and Treatment of Stroke (21, 22), with a confirmed diagnosis of ischemic stroke or hemorrhagic stroke via cranial computed tomography (CT) or magnetic resonance imaging (MRI); (2) Time from symptom onset to hospital admission ≤24 h, with subsequent admission to the NICU for intensive care lasting ≥72 h; (3) Aged ≥18 years; (4) Complete clinical data, including no missing key information on demographic characteristics, disease severity indicators, therapeutic interventions, and swallowing function assessment results; (5) Consciousness level permitting completion of swallowing function evaluation (Glasgow Coma Scale [GCS] score ≥6) or definitive determination of swallowing function status through objective instrumental examinations.

**Figure 1:**
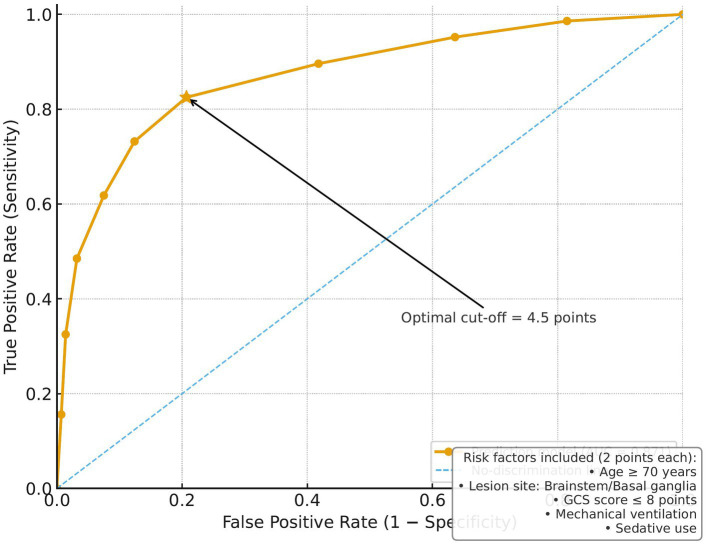
ROC curve for dysphagia prediction model in severe stroke patients.

Exclusion criteria: (1) Any documented history of dysphagia prior to the index stroke, irrespective of etiology; (2) Severe comorbidities that could independently affect swallowing function or confound outcome assessment (e.g., advanced liver failure, malignant tumors, uncorrected coagulopathy)—the specific INR cutoff of ≥1.5 was removed as it was considered arbitrary; (3) Voluntary discharge, transfer to another medical facility, or death during hospitalization due to critical illness, which precluded the completion of swallowing function assessment; (4) Incomplete clinical data with missing key influencing factors (e.g., lesion location, GCS score, mechanical ventilation status); (5) Comorbidity with mental illness or severe cognitive impairment, rendering the patient unable to cooperate with required clinical evaluations (Figure 1).

### Diagnostic criteria for dysphagia

The diagnostic criteria were comprehensively determined with reference to the *Chinese Guidelines for the Assessment and Intervention of Post-Stroke Dysphagia* (21), combining clinical symptoms, standardized assessments, and instrumental examination results: (1) Clinical symptoms: Choking, aspiration, delayed swallowing, difficulty in swallowing, food residue in the mouth, etc., during eating; (2) Standardized assessment: The Water Swallowing Test (30 mL warm water) was used for assessment, with a grade ≥III; (3) Instrumental examination: For patients with unclear or suspected dysphagia in clinical assessment, Fiberoptic Endoscopic Evaluation of Swallowing (FEES) or Video Fluoroscopic Swallow Study (VFSS) was performed to confirm the presence of abnormal movement of swallowing structures, bolus transport disorders, or evidence of aspiration. Meeting any of the above criteria was sufficient for a diagnosis of dysphagia.

### Data collection

Data were collected from January 2023 to August 2025 through the hospital’s electronic medical record system, NICU nursing record system, and laboratory examination database. The collected content strictly corresponded to the candidate influencing factors, including: (1) Demographic characteristics: Gender, age, BMI (calculated based on height and weight measured within 24 h after admission, BMI = weight kg/height m^2^); (2) Disease-related factors: Stroke type (ischemic/hemorrhagic, determined based on imaging examination results), lesion site—due to limited sample size for individual sub-sites (e.g., midbrain vs. pons vs. medulla), we used a binary classification: brainstem/basal ganglia vs. other sites, confirmed according to cranial CT or MRI reports; GCS score (the worst consciousness state score within 24 h after admission); (3) Treatment and intervention factors: Mechanical ventilation status (yes if invasive mechanical ventilation was received for ≥24 h; this cutoff was chosen to capture sustained exposure rather than brief peri-procedural use), sedative use (yes if sedatives such as benzodiazepines or propofol were used for ≥48 h; similarly, this threshold reflects sustained sedation beyond short-term intubation procedures); (4) Underlying diseases and complications: Hypertension (previously confirmed diagnosis or systolic blood pressure ≥140 mmHg and diastolic blood pressure ≥90 mmHg at admission), diabetes mellitus (previously confirmed diagnosis or fasting blood glucose ≥7.0 mmol/L; we did not differentiate between insulin-dependent and non-insulin-dependent diabetes subtypes, as this information was not systematically recorded in the retrospective database), pulmonary infection (diagnosed based on clinical symptoms such as fever, cough, and expectoration during hospitalization, combined with elevated white blood cell count in blood routine, inflammatory infiltration indicated by chest CT, and positive sputum culture results). After all data were extracted, they were entered into Excel 2019 to establish a database. A double-entry method was adopted for data entry, and cross-validation was performed after entry. Logical errors or missing data were promptly verified and supplemented by reviewing the original medical records to ensure the completeness and accuracy of the database.

### Statistical analysis

SPSS 26.0 statistical software was used for data processing and analysis, and GraphPad Prism 9.0 software was used for drawing ROC curves. All statistical tests were two-tailed, and a *p*-value <0.05 was considered statistically significant. Quantitative data were first tested for normality using the Shapiro–Wilk test. Normally distributed quantitative data were expressed as mean ± standard deviation, and intergroup comparisons were performed using the independent samples t-test; non-normally distributed quantitative data were expressed as median (interquartile range) [M (Q1, Q3)], and intergroup comparisons were performed using the Mann–Whitney U test. Categorical data were expressed as case number (percentage) [*n* (%)], and intergroup comparisons were performed using the *χ*^2^ test; when the theoretical frequency *T* < 5, Fisher’s exact probability test was used. Pearson correlation analysis (for bivariate normally distributed data) or Spearman rank correlation analysis (for non-normally distributed data or ordinal categorical variables) was used to explore the correlation between each candidate factor and the occurrence of dysphagia. Factors with *p* < 0.05 in univariate analysis were included in multivariate Logistic regression analysis (enter method, *α*-in = 0.05, *α*-out = 0.10) to identify the independent influencing factors for dysphagia in patients with severe stroke, and the odds ratio (OR) and 95% confidence interval (95%CI) of each factor were calculated. For the multivariate logistic regression, variables were coded as follows: dysphagia (yes = 1, no = 0); age (≥70 years = 1, <70 years = 0); lesion site (brainstem/basal ganglia = 1, others = 0); GCS score (≤8 points = 1, >8 points = 0); mechanical ventilation (yes = 1, no = 0); sedative use (yes = 1, no = 0). The Hosmer-Lemeshow test was used to evaluate the goodness-of-fit of the regression model (*p* > 0.05 indicated a good model fit), and the Nagelkerke *R*^2^ was used to test the explanatory power of the model. A predictive model was constructed based on the independent risk factors identified by multivariate Logistic regression analysis. ROC curves were drawn, and the area under the curve (AUC), sensitivity, specificity, and Youden index were calculated to evaluate the predictive value of the model for the risk of dysphagia in patients with severe stroke and determine the optimal predictive cutoff value.

## Results

### Patient characteristics and incidence of dysphagia

A total of 358 patients with severe stroke were enrolled in this study, among whom 288 developed dysphagia, resulting in an incidence rate of 80.45%. The diagnosis of dysphagia was made based on clinical symptoms alone in 62 patients (21.5%), by the Water Swallowing Test (grade ≥III) in 186 patients (64.6%), and by instrumental examination (FEES/VFSS) in 40 patients (13.9%). The median time from NICU admission to dysphagia assessment was 48 h (IQR: 36–72 h). Among patients with dysphagia, 231 (80.2%) required nasogastric tube feeding, and 37 (12.8%) received percutaneous endoscopic gastrostomy (PEG) during hospitalization.

Comparison of baseline data (Table 1) showed no statistically significant differences between the dysphagia group and the non-dysphagia group in terms of gender, BMI, stroke type, or medical history of hypertension and diabetes mellitus (all *p* > 0.05). Significant differences were observed between the two groups in age, lesion site, GCS score, mechanical ventilation use, sedative use, and pulmonary infection status (all *p* < 0.05). The dysphagia group had a higher proportion of patients aged ≥70 years (73.61% vs. 32.86%), a higher proportion with lesions located in the brainstem or basal ganglia (81.59% vs. 40.00%), lower GCS scores, and higher rates of mechanical ventilation and sedative use.

**Table 1:** Baseline data of severe stroke patients and comparison between groups (*n* = 358).

Variables	Dysphagia group (*n* = 288)	Non-dysphagia group (*n* = 70)	*p*
Gender, *n* (%)			0.666
Male	165 (57.3%)	42 (60.0%)	
Female	123 (42.7%)	28 (40.0%)	
Age (y), mean ± SD	73.52 ± 8.64	65.28 ± 7.95	<0.001
Age ≥70 y, *n* (%)	212 (73.6%)	23 (32.9%)	<0.001
BMI (kg/m^2^), mean ± SD	22.36 ± 3.15	23.12 ± 2.89	0.076
Stroke type, *n* (%)			0.071
Ischemic	203 (70.5%)	41 (58.6%)	
Hemorrhagic	85 (29.5%)	29 (41.4%)	
Lesion site, *n* (%)			0.001
Brainstem/basal ganglia	235 (81.6%)	28 (40.0%)	
Other sites	53 (18.4%)	42 (60.0%)	
GCS score (points), mean ± SD	8.25 ± 2.36	11.68 ± 1.85	0.031
Mechanical ventilation, *n* (%)	196 (68.1%)	18 (25.7%)	0.016
Sedative use, *n* (%)	172 (59.7%)	12 (17.1%)	<0.001
Hypertension, *n* (%)	198 (68.8%)	45 (64.3%)	0.417
Diabetes mellitus, *n* (%)	142 (49.3%)	29 (41.4%)	0.208
Pulmonary infection, *n* (%)	165 (57.3%)	21 (30.0%)	0.061

BMI = Body Mass Index; GCS = Glasgow Coma Scale. The t/χ^2^ column has been removed as requested.

### Univariate analysis and correlation

Correlation analysis results (Table 2) revealed that age, GCS score, lesion site, mechanical ventilation use, sedative use, and pulmonary infection were significantly correlated with the occurrence of dysphagia (all *p* < 0.05). GCS score showed a strong negative correlation with dysphagia (*r* = −0.623, *p* < 0.001). Age showed a moderate positive correlation (*r* = 0.486, *p* < 0.001), and mechanical ventilation use, sedative use, and lesion site also showed significant positive correlations (*r* = 0.426, 0.398, and 0.385, respectively; all *p* < 0.001). BMI, stroke type, hypertension, and diabetes mellitus showed no statistically significant correlations with dysphagia (all *p* > 0.05).

**Table 2:** Correlation analysis between dysphagia and related factors in severe stroke patients.

Variables	*r*	*p*
Age (years)	0.486	<0.001
BMI (kg/m^2^)	−0.098	0.076
GCS score (points)	−0.623	<0.001
Stroke type (Ischemic = 1, Hemorrhagic = 2)	−0.102	0.071
Lesion site (Brainstem/basal ganglia = 1, Others = 2)	0.385	<0.001
Mechanical ventilation (Yes = 1, No = 2)	0.426	<0.001
Sedative use (Yes = 1, No = 2)	0.398	<0.001
Hypertension (Yes = 1, No = 2)	0.058	0.417
Diabetes mellitus (Yes = 1, No = 2)	0.086	0.208
Pulmonary infection (Yes = 1, No = 2)	0.256	<0.001

BMI = Body Mass Index; GCS = Glasgow Coma Scale.

### Multivariate logistic regression

Factors with *p* < 0.05 in univariate analysis were included in the multivariate Logistic regression model. Variable coding is described in the Statistical Analysis subsection of the Methods. The results (Table 3) indicated that age ≥70 years, lesion site in the brainstem/basal ganglia, GCS score ≤8 points, mechanical ventilation use, and sedative use were independent risk factors for dysphagia (all *p* < 0.001). Patients with a GCS score ≤8 points had the highest risk (OR = 8.654, 95%CI: 5.012–14.928), followed by those receiving mechanical ventilation (OR = 6.863, 95%CI: 4.015–11.729) and those aged ≥70 years (OR = 6.452, 95%CI: 3.825–10.936). Lesion site in the brainstem/basal ganglia (OR = 4.608, 95%CI: 2.751–7.723) and sedative use (OR = 5.382, 95%CI: 3.156–9.187) also significantly increased the risk. The Hosmer-Lemeshow test showed *χ*^2^ = 6.258, *p* = 0.628, indicating good model fit. Nagelkerke *R*^2^ was 0.683.

**Table 3:** Multivariate logistic regression analysis of independent risk factors for dysphagia.

Variables	OR	95% CI	*p*
Age ≥70 years	6.452	3.825–10.936	<0.001
Lesion site (brainstem/basal ganglia)	4.608	2.751–7.723	<0.001
GCS score ≤8 points	8.654	5.012–14.928	<0.001
Mechanical ventilation	6.863	4.015–11.729	<0.001
Sedative use	5.382	3.156–9.187	<0.001

Nagelkerke *R*^2^ = 0.683; Hosmer-Lemeshow test *χ*^2^ = 6.258, *p* = 0.628. OR = Odds Ratio; CI = Confidence Interval; GCS = Glasgow Coma Scale. The *β* and Wald columns have been removed for conciseness.

### Predictive model performance

A dysphagia prediction model was constructed based on the five independent risk factors. The area under the ROC curve (AUC) was 0.864 (95%CI, 0.812–0.916, *p* < 0.001). Because the scoring system assigns 0 or 2 points per factor (total range 0–10, integer steps of 2), the optimal integer cutoff for clinical use was determined as a total score ≥6 points (corresponding to the ROC-derived threshold of 4.5 points). At this cutoff, sensitivity was 73.2%, specificity was 87.6%, and the Youden index was 0.608 (Table 4).

**Table 4:** Sensitivity, specificity and Youden index of the dysphagia prediction model at different score thresholds.

Total score	Sensitivity	Specificity	Youden index
0.5	1.000	0.000	0.000
1.5	0.986	0.185	0.171
2.5	0.952	0.364	0.316
3.5	0.896	0.582	0.478
4.5	0.825	0.793	0.618
5.5	0.732	0.876	0.608
6.5	0.618	0.925	0.543
7.5	0.485	0.968	0.453
8.5	0.325	0.986	0.311
9.5	0.156	0.993	0.149
10.5	0.000	1.000	0.000

The scoring system assigns 2 points to each of the five risk factors (age ≥70 years, brainstem/basal ganglia lesion, GCS score ≤8 points, mechanical ventilation, sedative use). Total score range: 0–10 (integer steps of 2). The ROC-derived optimal cutoff was 4.5 points; the corresponding integer cutoff for clinical use is total score ≥6 points (sensitivity 73.2%, specificity 87.6%, Youden index 0.608). GCS = Glasgow Coma Scale.

The scoring system assigns 2 points to each independent risk factor: age ≥70 years, lesion site in brainstem/basal ganglia, GCS score ≤8 points, mechanical ventilation use, and sedative use. Higher total scores indicate higher risk of dysphagia.

## Discussion

### Overall occurrence characteristics of dysphagia in patients with severe stroke

The incidence of dysphagia in this cohort was 80.45%, which is higher than the reported prevalence of 42–80% in general stroke populations (12). This high incidence likely reflects the severity of illness in our NICU cohort, as all enrolled patients had severe neurological deficits and impaired consciousness. Swallowing function depends on an intact neuromuscular pathway, and central nervous system damage from stroke directly disrupts the swallowing reflex (22). In addition, invasive treatments such as mechanical ventilation and sedation may further increase dysphagia risk (23, 24). These findings confirm that dysphagia is a very common complication in severe stroke, highlighting the need for early assessment and intervention.

### Independent influencing factors of dysphagia

Multivariate analysis identified five independent risk factors: age ≥70 years, brainstem/basal ganglia lesion location, GCS score ≤8, mechanical ventilation, and sedative use. These findings are generally consistent with prior studies, though our analysis focused specifically on critically ill patients.

Age is a non-modifiable factor, possibly through age-related decline in laryngopharyngeal muscle function and neural reflex sensitivity. Lesion location—the brainstem contains the central swallowing pattern generator, and the basal ganglia are involved in motor control of swallowing (25, 26). In this study, 81.6% of dysphagic patients had lesions in these regions, supporting their critical role. GCS score ≤8 indicates severe consciousness impairment; an intact level of consciousness is necessary to initiate the swallowing reflex, and patients with reduced consciousness cannot coordinate swallowing, raising aspiration risk (27). This is consistent with the strong negative correlation we observed (*r* = −0.623).

Mechanical ventilation and sedative use are clinical interventions that may adversely affect swallowing. Endotracheal intubation or tracheostomy can directly impair laryngopharyngeal structure and function, while sedatives suppress central nervous system activity, reduce pharyngeal muscle tone, and prolong swallowing latency (28). Both factors had elevated rates in the dysphagia group (68.1 and 59.7%, respectively) and high ORs (6.863 and 5.382). These results suggest that clinicians should carefully balance treatment benefits against dysphagia risk, though causality cannot be firmly established from this retrospective design. Factors such as gender, BMI, hypertension, and diabetes mellitus showed no significant associations. This may be because the effects of chronic comorbidities are overshadowed by acute severe neurological damage in this population (29).

### Predictive model performance and clinical utility

The model based on the five risk factors achieved an AUC of 0.864. At the ROC-derived cutoff of 4.5 points (corresponding to an integer score ≥6 in the simplified scoring system), sensitivity was 73.2% and specificity 87.6%. However, for screening purposes, a lower threshold (e.g., total score ≥4 points, sensitivity 89.6%) may be preferred to maximize detection of at-risk patients. The model uses routine NICU data and requires no additional testing; it can be calculated quickly at the bedside. Nevertheless, this model should be considered a risk-stratification tool, not a substitute for diagnostic swallowing assessments such as FEES (17). For patients with impaired consciousness who cannot cooperate with standard tests, the model may provide an early warning to guide further evaluation (30). The Nagelkerke *R*^2^ was 0.683, indicating that the model explains 68.3% of the variance in dysphagia occurrence. Although the model requires external validation before clinical implementation, it shows potential to assist in risk stratification and early warning for dysphagia in severe stroke patients.

### Comparison with previous literature

Our core findings align with most prior reports, but some differences exist. Previous studies have suggested that diabetes mellitus may increase dysphagia risk (31, 32), but we did not find a significant association. This discrepancy may be due to our sample size or the predominance of acute neurological injury over chronic diabetic effects in severe stroke. Additionally, our model uses only readily available clinical variables, in contrast to some models that include nutritional or laboratory parameters, which may enhance practicality in critical care settings. These differences underscore that dysphagia risk factors are population-specific, and management protocols for non-severe stroke should not be directly applied to severe stroke patients without validation.

### Limitations

This study has several limitations. First, the single-center retrospective design may introduce selection bias, and the results require validation in multicenter prospective cohorts. Second, the diagnosis of dysphagia relied primarily on clinical symptoms and the Water Swallowing Test; many patients with impaired consciousness could not undergo gold-standard FEES or VFSS, potentially affecting diagnostic accuracy. Third, we used a binary classification for lesion site (brainstem/basal ganglia vs. other) because the sample size was insufficient for more granular sub-site analysis (e.g., midbrain vs. pons vs. medulla). Fourth, important clinical variables such as NIHSS score, aphasia, dysarthria, and facial droop were not systematically recorded in our retrospective database and could not be included. Fifth, we did not differentiate between insulin-dependent and non-insulin-dependent diabetes subtypes. Sixth, the cutoffs for mechanical ventilation (≥24 h) and sedative use (≥48 h) were arbitrary, chosen to capture sustained exposure; sensitivity analyses using different thresholds were not performed. Seventh, GCS score ≤8, mechanical ventilation, and sedative use are clinically interrelated, and multicollinearity may have inflated the odds ratios; a simplified model using fewer of these variables might perform similarly and should be explored in future studies. Finally, pulmonary infection was originally considered as a candidate factor but was removed because it is more likely a consequence rather than a predictor of dysphagia. We also did not assess the effectiveness of interventions guided by the model, so its impact on patient outcomes remains unknown.

### Future directions

Future research should include multicenter prospective cohort studies with larger sample sizes to validate and refine the model. Additional predictor variables (e.g., NIHSS, aphasia, instrumental swallowing measures) should be incorporated. Randomized controlled trials are needed to determine whether model-guided stratified intervention reduces dysphagia-related complications and improves long-term functional outcomes. Finally, artificial intelligence approaches integrating imaging and physiological data could enhance prediction accuracy and timeliness.

## Conclusion

In this cohort of patients with severe stroke, the incidence of dysphagia was 80.45%, with age ≥70 years, brainstem/basal ganglia lesion location, GCS score ≤8, mechanical ventilation, and sedative use identified as independent risk factors. A predictive model incorporating these five factors showed good discrimination (AUC = 0.864), though the optimal cutoff requires refinement for clinical use. These findings provide an evidence-based basis for early risk stratification, but the model’s generalizability is limited by the single-center, retrospective design. External validation in multicenter prospective studies is needed before the model can be recommended for routine clinical application.

## Data Availability

The raw data supporting the conclusions of this article will be made available by the authors, without undue reservation.
